# Roles, Barriers, and Recommendations for Community Health Workers Providing Community-Based HIV Care in Sub-Saharan Africa: A Review

**DOI:** 10.1089/apc.2022.0020

**Published:** 2022-04-14

**Authors:** Sanele Ngcobo, Susan Scheepers, Nothando Mbatha, Estelle Grobler, Theresa Rossouw

**Affiliations:** ^1^ Department of Family Medicine, University of Pretoria, Pretoria, South Africa.; ^2^ Medical Library, University of Pretoria, Pretoria, South Africa.; ^3^ Department of Public Health and University of Pretoria, Pretoria, South Africa.; ^4^ Department of Immunology, University of Pretoria, Pretoria, South Africa.

**Keywords:** HIV, Community Health Workers, roles, barriers, antiretroviral, stigma

## Abstract

While the impact of Community Health Workers (CHWs) on home-based human immunodeficiency virus (HIV) care has been documented, barriers and recommendations have not been systematically reviewed. Following the reporting requirements of the Preferred Reporting Items for Systematic Reviews and Meta-Analyses (PRISMA) guidelines, we used an aggregative narrative synthesis approach to summarize the results of qualitative studies published between January 1, 2000, and November 6, 2020 in the following databases: PubMed, CINAHL, PsychINFO, Web of Science, and Google Scholar. In total, 17 studies met the selection criteria and were included in the analysis. They reported on a range of roles played by CHWs in HIV care, including education and health promotion; HIV-specific care (HIV testing services; screening for opportunistic infections and acute illness); medication delivery; tracing persons who had defaulted from care; and support (treatment support; referral; home-based care; and psychosocial support).

Many different barriers to community-based HIV care were reported and centered on the following themes: Stigma and nondisclosure; inadequate support (lack of resources, inadequate training, inadequate funding, and inadequate monitoring); and health system challenges (patients' preference for more frequent visits and poor integration of CHWs in the wider health care system). Recommendations to mitigate these barriers included: addressing HIV-related stigma; introducing updated and relevant CHW training; strengthening the supervision of CHWs; coordinating care between the home and facilities; incorporating patient-centered mHealth approaches; and committing to the funding and resources needed for successful community-based care. In summary, CHWs are providing a variety of important community-based HIV services but face challenges with regards to training, resources, and supervision.

## Introduction

An estimated 37.7 million (30.2–45.1 million) people were living with the human immunodeficiency virus (HIV) at the end of 2020.^1^ About 5000 new HIV infections are reported on a daily basis globally, 61% of which occur in sub-Saharan Africa (SSA).^2^ Antiretroviral therapy (ART) increases life expectancy: a 2017 meta-analysis reported that starting ART at age 20 years increases life expectancy by an additional 22.9 years [95% confidence interval (CI) 18.4–27.5 years] for men and 33.0 years (95% CI 30.4–35.6 years) for women living in low- and middle-income countries (LMIC). Despite this benefit, uptake and persistence have been suboptimal in LMIC.^3^ Of the ±20.6 million (95% CI 16.8–24.4 million) people living with HIV (PLWHIV) in Eastern and Southern Africa, only 78% were accessing ART in 2020.^1^ Without innovative interventions, these regions will struggle to attain the United Nations Program on HIV/AIDS (UNAIDS) 95 95 95 goals by 2030.^4^

Specifically, the second goal of having all people diagnosed with HIV infection on sustained ART^5^ remains an obstacle in SSA. A 2007 systematic review reported that fewer than two-thirds of PLWHIV in SSA remained on treatment 2 years after ART initiation.^6^ This is supported by more recent studies, which have shown that only 70%, 65%, and 63% of people remained on treatment after 2, 3, and 6 years of ART, respectively.^7–9^ Community Health Workers (CHWs) have emerged as a valuable health care cadre to manage and support the rising number of people who need to be initiated and maintained on ART.^10^

CHWs receive job-related training in the context of specific interventions, but have no “formal professional, certificated, or degreed tertiary education.”^11^ These individuals are selected on the basis of their in-depth understanding of a community's culture and language, and their primary goal is to provide culturally appropriate primary health care services to the community.^12^ They generally work with the underserved and are mostly indigenous to the community in which they work, not only in terms of ethnicity and language, but also in terms of shared socioeconomical and structural experiences.^13^ In low-resource settings, CHWs are frontline health care providers who shoulder much of the health service delivery burden. CHW programs have been introduced in many SSA countries in an effort to expand HIV care to communities.^14–16^ In addition, some have argued that more HIV care responsibilities should be shifted from doctors to nurses and CHWs.^17^ While task shifting as well as the role and the impact of CHWs in HIV care in SSA have been documented and reviewed,^16,18,19^ the barriers to and recommendations for the delivery of such care in the community have not been systematically reviewed.

## Methods

We performed a systematic, aggregated, narrative review using evidence from qualitative studies according to an established methodology^20^ and followed the reporting requirements of the preferred reporting items for systematic reviews and meta-analyses (PRISMA) guidelines.^21^

### Definition

For the purposes of this review, a CHW was defined as “any health worker who performs functions related to health care delivery at a community level; has trained in some way in the context of the intervention; and has no formal professional or paraprofessional certificate or degree in tertiary education.”^22^

### Search strategy and study selection

We searched the following databases for articles published in English between January 1, 2000 and November 6, 2020: PubMed, CINAHL, PsychINFO, and Web of Science. Google Scholar was used to identify articles that might have been missed in the other four databases. The initial search strategy was developed by the principal investigator in consultation with the librarian (S.S.). A second librarian (E.G.) reviewed and approved the search strategy before the screening process started. Initially, the following search words were used: barriers OR obstacles OR challenges OR difficulties OR issues OR problems AND Community Health Workers AND HIV care. Only 280 articles were found, and the search strategy was then broadened to also include the following MeSH words: “HIV AND Community Health Workers” in PubMed, CINAHL, PsychINFO, and Web of Science.

### Data extraction and analysis

**FIG. 1.**
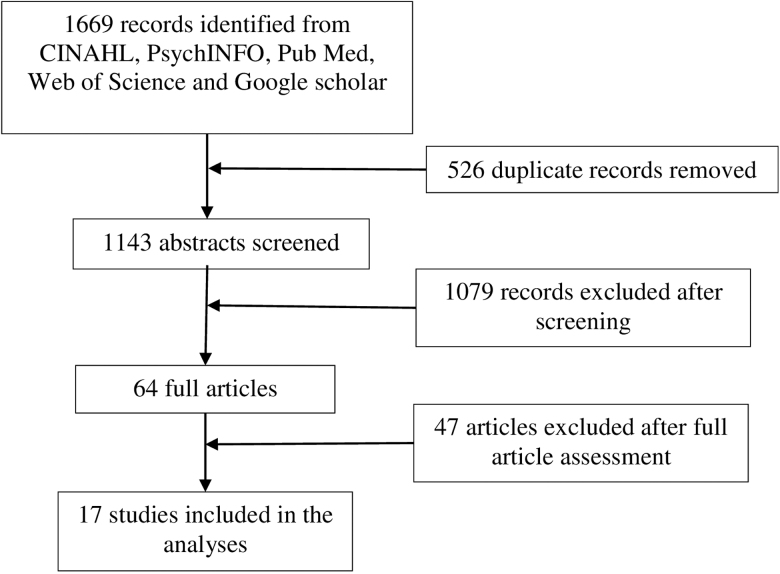
Schematic representation of study selection.

A total of 1669 articles were exported directly from different databases to EndNote X8. A total of 526 duplicates were identified and excluded. S.N. screened abstracts of 1143 using pre-defined screening criteria. Studies were included if they clearly documented the roles, barriers, or recommendations of CHWs providing community-based HIV care in SSA. After full text screening of 64 articles, S.N. and N.M. individually selected a total of 19 articles. Minor discrepancies in screening decisions were identified and resolved through consensus (by S.N. and N.M.): two studies were further excluded; hence, 17 articles were included in the final analysis (Fig. 1). The quality and relevance of all articles were reviewed by S.N. and N.M., using the CASP tool (Supplementary Table S1).^23^ A thematic analysis approach was used to produce rudimentary synthesis of findings across all 17 articles. Accordingly, all 17 articles were imported into ATLAS.ti 8 (Windows XP). Codes, ideas, and concepts were identified, connections identified, and brought together to form different themes.

### Inclusion and exclusion criteria

Qualitative or mixed method studies from SSA, which reported on the roles, barriers, and/or recommendations for offering community-based HIV care by CHWs between January 1, 2000 and November 6, 2020 were included in this study. Only studies published in English were included. Articles without qualitative components were excluded. Studies with HIV-positive participants who were receiving or observing home-based HIV care services were included. Studies with CHWs and other health care workers as participants who were providing or observing home-based HIV care services were included.

### Ethics approval and consent to participate

Since this is a review article, informed consent was not required. The study forms part of a larger study that was approved by the Research Ethics Committee of the Faculty of Health Sciences, University of Pretoria (reference No.: 580/2018).

## Results

The sample sizes of the 17 studies, which met the inclusion criteria, ranged from 19 to 393. Participants were from South Africa (10),^24–33^ Kenya (2),^34,35^ Uganda (2),^36,37^ Malawi (1),^38^ Zambia (1),^39^ Ethiopia (1),^37^ Lesotho (1),^40^ Mozambique (1),^41^ Zimbabwe (1),^42^ and eSwatini (1).^33^ One study was performed in four countries within the southern African region, namely Lesotho, Mozambique, South Africa, and eSwatini.^33^ A summary and narrative synthesis of the 17 included studies are provided in Table 1. The description includes the name of the country where the study was performed, the methodology, sample size, study participants, role of CHWs, barriers to providing community-based care, and recommendations to improve such care. This summary was extracted from each of the 17 articles by S.N. and reviewed by T.R.

**Table 1.** Summary of Included Studies and Narrative Synthesis

No.	Articles	Setting	Methodology	Sample size	Study participants	Role of CHWs	Barriers	Recommendations
(1)	Suri et al.^24^	South Africa (KZN)	Mixed methods (survey and FGD)	135	CHWs—120, regional coordinators—2, doctors—2, nurses—3, NGO staff—4, RC—3, DDG—1	Curtail HIV transmission through education and counseling	Lack of resources for CHW	Improve CHWs' accountability, monitoring, and availability of resources and support
(2)	Rachlis et al.^34^	Western Kenya	Exploratory qualitative study (IDI and FGD)	288	PLWHIV—110, TB patients—39, HTN patients—21, caregivers—24, community leaders—10, HCWs—34	Promote primary health care and generate awareness about relevant health issues	Poor understanding of confidentiality; lack of CHW information on relevant health issues; nonintentional disclosure of HIV status; lack of formal training	Recruit well-respected members of the community as CHWs; offer comprehensive training; provide informative pamphlets to CHWs for distribution to households
(3)	Nxumalo et al.^25^	South Africa (Gauteng and Eastern Cape)	Comparative qualitative study (FGD, IDI and observations of CHWs)	74	CHWs, government representatives and policy makers^a^	Deliver medication; trace patients who have defaulted from ART; HIV-related health education	Lack of support; lack of trust by household members	Strengthen the links between sectors and departments at different levels of government
(4)	Naidoo et al.^26^	South Africa	Qualitative exploratory study (IDI and FGD)	101	CHWs—11, nurses—12, community leaders—21, social workers —10, community members—38, OTL—9	Health education on HIV prevention; screening for HIV; adherence support and trace patients who defaulted; identify individuals with deteriorating health while on ART	Clients' denial of HIV status and fear of stigma; resource limitations and inadequate integration into the primary health care system and workflows	Integrate CHWs into the primary health care system; formalize ward-based outreach teams; increase CHW stipends
(5)	Loeliger et al.^27^	South Africa (KZN)	Qualitative study (FGD)	21	CHWs—21	Health education; counseling and treatment support; linkage to health care and social services	Insufficient patient education and social support; denialism; patient dissatisfaction with health care services; men prohibiting wives or children from taking ART	Equip CHWs with resources and knowledge to be able to provide HIV-related support to their patients; prioritize patient-centered care; collaborate with traditional healers and church leaders; deal with socioeconomic factors
(6)	Ngcobo and Rossouw^28^	South Africa (Gauteng)	Qualitative study (FGD)	58	CHWs—25, OTL—11, PLWHIV—22	HIV counseling and testing; trace patients who defaulted	Incorrect addresses; fear of stigma through association with CHWs (especially those in uniform); little or no preparation of patients for HBC; lack of confidentiality and trust	Integrate CHWs into clinics and existing support structures; improve training on confidentiality and HIV testing for CHWs; rethink the recruitment, scope of work and safety of CHWs as well as the requirement for patients' identification numbers
(7)	Heunis et al.^29^	South Africa	Qualitative study (FGD)	110	CHWs—57, lay counselors—40, TB and HIV program managers—13	Treatment support	Perceived lack of confidentiality; lack of information, education, and communication materials provided in local languages	Improve HTS; provide HTS-related training
(8)	Wanga et al.^35^	Kenya	Qualitative study (IDI and FGD)	70	PLWHIV—40, HCWs—30	Education and support for HIV and PMTCT; linking and referral to facilities	Potential breach of confidentiality by CHWs; inadvertent disclosure of HIV status; lack of private meeting places in clients' homes; potential stigmatization and social isolation of CHWs in the communities	Gain trust and provide information not always possible in a more formal provider–patient relationship
(9)	van Heerden et al.^30^	South Africa	Qualitative study (FGD and IDI)	37	Home-based HTS field staff—10, CHWs—12, PLWHIV—10, health officials—5	Conduct home visits, give health education, conduct health assessment, and refer household members (to clinic) who need medical attention	Lack of communication and sharing of patient health information between clinics, between clinics and CHWs, and between clinics and patients; lack of trust between CHWs and facility staff	Introduce biometrics to improve patient identification
(10)	Pellecchia et al.^38^	Malawi	Qualitative study (FGD, IDI, and participant observation)	94	CHWs—60, PLWHIV—9, medical assistant—5, nurses—2, health surveillance assistants—18	Adherence support; emotional support; income-generating activities	Stigma; unwillingness to disclose HIV status; change of residence	Bigger investment in community models of care, supported by strong networks of PLWHIV
(11)	Magidson et al.^31^	South Africa	Qualitative study (semistructured qualitative interviews)	30	HIV and AOD treatment staff, and PLWHIV with moderate, problematic AOD^a^	Detect substance use problems among some PLWHIV	Little formal training in screening and interventions for problematic AOD use among PLWHIV; CHWs may perpetuate inaccurate messaging to patients about not mixing alcohol and ARVs	Additional training on problematic AOD use; accurate information about the risk of mixing AOD and ART
(12)	Mottiar and Lodge^32^	South Africa	Qualitative study (FGD, questionnaire and IDI)	60	CHWs—55, leaders from AIDS organizations—5	Identify patients with unknown HIV status and link them to HST services; treatment support, including adherence support; home delivery of ART; trace persons who have defaulted; help with facility-based duties, including screening for TB, measure vital signs, etc.	Stigmatization of PLWHIV; fear of stigmatization; unsustainability of CHWs intervention	Direct supervision of CHWs by clinical staff members
(13)	Busza et al.^42^	Zimbabwe	Qualitative study (longitudinal semistructured interviews)	19	CHWs—19	Deliver social support; adherence support	Lack of intensive supervision and mentoring; fear of stigmatization among PLWHIV when CHWs wear a uniform	CHWs should not wear uniforms when this is the preference of the PLWHIV they visit
(14)	Alamo et al.^36^	Uganda	Qualitative study (IDI and semistructured interviews)	393	PLWHIV—347, CHWs—46	Basic counseling; medication adherence; TB and HIV treatment; referral for medication side effects; record keeping	Workload; poor performance by CHWs; lack of trust; bad attitude by CHWs; nondisclosure of HIV status to CHWs	Regularly assess performance and attitudes of CHWs; integrate CHW activities into the broader health system
(15)	Gusdal et al.^37^	Uganda and Ethiopia	Qualitative study (semistructured interviews)	118	Patients—79, peer counselors —17, providers in ART facilities—22	Treatment adherence support, HIV awareness	Inadequate supervision; working too few hours a day; frustration and emotional agony when CHWs observed sick PLWHIV not initiated on ART due to lack of medicines; narrow geographic scope; fear of stigma by PLWHIV when traced by CHW after defaulting treatment; unwillingness of PLWHIV to provide correct addresses and phone numbers	Develop a support structure for CHWs; formal recognition and regulation of CHWs
(16)	Cataldo et al. (2015)^39^	Zambia	Qualitative study (IDI and observations of CHWs)	104	Staff from 3 local HBC organizations—17, ART clinic staff—8, home-based caregivers—48, HBC clients—31	Treatment adherence support; trace people who have defaulted; deliver medication; screen for opportunistic infections and ART side effects	Lack of formal training and recognition; poor remuneration; nondisclosure of HIV status; some PLWHIV expect food and financial support from CHWs: if they are unable to provide these, their relationship with PLWHIV suffers	Professionalization of CHW program; make the necessary specialized training available; strengthen supervisory support
(17)	De Neve et al.^40^	Lesotho, Mozambique, South Africa, and eSwatini	A four-country qualitative study (semistructured interviews)	60	Donors, government officials, and expert observers involved in CHW programs^a^	Health education, HST, HBC, and ART adherence support	Highly fragmented care and poorly integrated into national health systems; wide range of stakeholders; lack of long-term support; HIV-specific approach, neglecting other conditions; unbalanced demographic profile of CHWs	Officially recognize CHW programs; standardize CHW training, incentives, and services; involve the community in decision making; provide adequate and long-term resources for CHW programs for HIV; move away from an HIV-specific approach toward comprehensive care

^a^ Number of participants per subcategory not reported.

AOD, alcohol and other drugs; ART, antiretroviral therapy; ARVs, antiretroviral medication; CHW, Community Health Worker; DDG, Deputy Director General; FGD, focus group discussion; HBC, home-based care; HCWs, health care workers; HIV, human immunodeficiency virus; HTN, hypertension; HTS, HIV testing services; IDI, in-depth interviews; KZN, KwaZulu Natal; NGO, nongovernmental organization; OTL, operational team leader; PLWHIV, people living with HIV; PMTCT, prevention of mother-to-child transmission; RC, research coordinator; TB, tuberculosis.

### Roles of CHWs

Table 2 provides a breakdown of the different roles that have been reported for CHWs while providing community-based HIV care. These roles can be categorized into three different themes: (1) Education and health promotion; (2) HIV-specific care: HIV testing services (HTS); screening for opportunistic infections and acute illness; medication delivery; tracing persons who had defaulted from care; and (3) Support: treatment support; referral; home-based care; and psychosocial support.^24–26,29,34,35,43–45^

**Table 2.** Roles of Community Health Workers in Community-Based HIV Care

Themes	Sub themes	Activities
Education and health promotion		General health education^34,40^ HIV/AIDS-related education^25,26^ HIV prevention^24,26,34,35,41^ Encourage males to undergo medical male circumcision^32^ Nutrition education^35^ Living with HIV^35,36^ HIV status disclosure^35,37,47^ HIV stigma^35^ ART literacy^35,36,45^ HIV comorbidities and coinfections^32,36^
HIV-specific care	HTS	Identify clients who need HTS and provide HTS^36–47^
	Screening	Screen for opportunistic infections, such as TB^24,29,32,34,36^ Screen for acute illnesses^36^
	Medication delivery and monitoring	Deliver ART for patients who are unable to reach clinics^25,32,38,40,48^ Perform pill count of ARVs^42,49^
	Trace ART defaulters	Trace patients who have been lost to follow-up or those who have missed their appointments^25–36^
Support	Treatment support	Support patients on ART and other chronic medication^26,27,29,35,36^ Remind patients to collect medication^50^ Motivate patients to take their medication^29,34^ Screen for treatment-related side effects^32,45^ Support women on PMTCT programs^35^
	Referral	Refer individuals who test positive for HIV^27^ Refer patients with ART side effects^36^ Refer individuals who screened positive for opportunistic infections^26^ Refer patients with acute illnesses^36^
	Home-based care and other activities	Cook for and feed patients^26,32,39^ Clean patients^25,26,39^ Change soiled linen^32^ Fetch water^26^ Raise funds to feed PLWHIV^32^
	Psychosocial support	Provide psychosocial support^25,26,34–36,39,42^ Develop coping strategies^35^ Linkage to social services^27^ Marital counseling ^35^

ART, antiretroviral therapy; ARVs, antiretroviral medication; HIV, human immunodeficiency virus; HTS, HIV testing services; PLWHIV, people living with HIV; PMTCT, prevention of mother-to-child transmission; TB, tuberculosis.

The first theme, education and health promotion, was a major focus of some articles and encompassed general health education as well as HIV-specific education, such as information about living with HIV, ART, and its side effects, as well as HIV coinfections, such as tuberculosis (TB). In this educational role, CHWs built on their knowledge acquired during facility-based HIV/ART counseling and literacy classes.^27^ Another major role fulfilled by CHWs was HIV-specific care across the HIV treatment cascade: from diagnosis to monitoring treatment adherence, screening for opportunistic infections and acute illness and tracing patients who have missed their clinic appointments, and those who have become lost to follow-up.^26,34,35^ Some CHWs were also doing home delivery of medication for individuals unable to travel to the clinic.^25,39,46^ Treatment support ranged from screening for treatment-related side effects, to reminders to collect treatment and motivating patients to remain adherent to treatment.^35,38,44,45^ CHWs were also responsible for referring all individuals who tested HIV positive, screened positive for opportunistic infections or acute illnesses, or ART side effects to health facilities.^36^

In one study, CHWs were playing an important role in supporting women on prevention of mother-to-child transmission (PMTCT) programs.^35^ Multiple studies reported that CHWs, supported by social workers, nurses, and clinicians, were providing psychosocial support, including marital counseling and teaching coping strategies.^25,26,34–36,39,42^ Some studies reported that CHWs even provided general home-based support, such as household cleaning for child-headed households affected by HIV^25^ and activities such as cooking for and feeding patients,^34,36,38^ cleaning, and fetching water for sick individuals unable to perform these activities themselves.^26^

### Barriers

Barriers to delivering community-based HIV services were divided into two distinct themes: (1) Patient-level barriers: stigma; nondisclosure of HIV status; concerns about confidentiality; patients' preference for more frequent visits; and (2) CHW- and health care system-level barriers: lack of resources; inadequate training; lack of support from relevant stakeholders; poor communication; inadequate funding; inadequate monitoring; and poor integration of CHWs into the health care system.

#### Patient-level barriers

##### Stigma

Patients' concern about real or perceived stigma was almost universally documented as a barrier to CHWs delivering HIV-related care in communities. Stigma was identified at various levels: from the household to the community and up to the facility level, and involved a wide spectrum of people, namely family members, community members, and health workers.^24–26,30^ In many studies, CHWs were involved in all these different levels, and stigma, therefore, affected them in many ways. Stigma manifested as low acceptance of HTS,^29^ HIV status nondisclosure to CHWs,^44^ and patients attending facilities far from their communities and providing incorrect addresses in those clinics.^26,28,37^ Three studies documented that stigma was worse when a CHW came from the local community.^28,35,44^ Conversely, some thought that CHWs from the community with the same ethnicity and language would have shared socioeconomical and structural experiences and therefore understand community members better.

##### Nondisclosure of HIV status

Many studies documented issues around disclosure. Patients feared unintentional disclosure of their HIV status since it is well known in the communities that HIV-infected persons are regularly visited by CHWs in their households.^24,26–30,34–36,38,44^ Some patients therefore elected not to disclose their HIV status to CHWs, which regrettably also made it impossible for CHWs to support them.^28^ Nondisclosure was also reported to be associated with the fear of stigmatization, in particular when CHWs came from the local community and thus shared the same social spaces as patients.^34,47^ Another reason PLWHIV feared unintentional disclosure was the lack of privacy in the household, which compromised confidentiality.^35^ While such individuals were willing to disclose to CHWs, they did not want their family members to know their status^26^ and some therefore elected not to be visited in their home but rather in a different location.^36^ A negative attitude by CHWs toward PLWHIV was also reported to negatively influence disclosure.^36^ Alamo et al. argued that negative attitude could possibly be linked to the pre-existing relationship between CHWs and PLWHIV.^36^

##### Concerns about confidentiality

Perceived lack of confidentiality was reported as a barrier and had a negative impact on disclosure and trust.^24,29,34,35^ According to Heunis et al., confidentiality was not a problem limited to CHWs; it also extended to the facility level where nurses and lay counselors test and manage PLWHIV. Confidentiality is even more important for CHWs staying in the same communities that they serve. Since they work with people they share social spaces with and constantly interact with their families and neighbors, confidentiality is an ethical and practical imperative.^34^ In an Ugandan study, CHWs staying in the same village as the PLWHIV they served were rated significantly worse than those not staying in the same village.^36^ The age of CHWs was also seen as an important factor, with some study participants fearing that younger people were not good at keeping private information secret.^34^ Another factor that came into play was CHWs' own HIV status: some CHWs felt that it was more difficult to gain the trust of patients when they were themselves uninfected and they believed that this negatively affected disclosure.^28^

##### Patient preference for more frequent visits

In contrast to the Alamo et al. study^36^ where many participants did not prefer home visits, a study by van Heerden et al.^30^ reported that PLWHIV were more comfortable with home than clinic visits since the conversations at home were more confidential and less stigmatizing. Patients preferred fewer, short visits since more frequent visits interfered with their lives and daily routines.^36^ More frequent visits to stable patients will, however, take time away from patients in greater need of CHW services and needs careful consideration.^36^

#### CHW-level and health care system barriers

##### Lack of resources

Lack of resources was frequently reported as a barrier to offering community-based HIV care by CHWs. These resources included educational materials, such as posters, flyers, pamphlets, and brochures that CHWs could distribute among community members, but also extended further to the equipment needed to perform their duties. In studies performed by Suri et al.^24^ and Naidoo et al.,^26^ CHWs complained that they did not have personal protective equipment like aprons, masks, and gloves, which made it difficult to help some patients. Community members appreciated the availability of equipment, such as sphygmomanometers and hemoglucometers, and this improved the communities' acceptance of CHWs.^26^ Unfortunately, most CHWs did not have such equipment.

In addition, CHWs sometimes ended up using their own money to pay for work-related expenses, such as stationary, travel, and airtime, with which to communicate with patients.^26^ Another resource that was frequently lacking was adequate space in health facilities, making it difficult for CHWs to conduct meetings and training sessions.^26^ All these resource challenges hindered their ability to meet the communities' needs with the consequence that patients and households did not derive the optimum benefit from these potentially valuable services.^25^

##### Inadequate training

While Naidoo et al.^26^ reported that CHWs were happy with the initial training they had received, other studies found that CHWs requested more training, especially with regard to HIV.^28,29,36^ Training should, however, not just be limited to HIV, since PLWHIV may present with many other physical and psychological health challenges. For instance, one study found that CHWs had not received adequate training to deal with PLWHIV using alcohol and other drugs.^44^ Importantly, CHWs reported that their low level of training negatively affected their rating by patients.^36^

##### Lack of support from relevant stakeholders

For a community program to be successful, there is a need for buy-in from stakeholders such as departments of health, nongovernmental organizations (NGOs), facility staff, and facility managers.^26^ Across SSA, it was reported that relevant stakeholders were not supporting CHWs adequately.^25,26^ When visiting households of PLWHIV, CHWs came across multifaceted challenges (social, clinical, financial etc.) requiring their intervention; without strong multidisciplinary and institutional support, they are unable to deal with these challenges.^25^ The perceived lack of support extended to emotional and moral support, given the strain of dealing with HIV-infected and, sometimes, dying patients.^24^

##### Poor communication

Lack of communication was frequently reported as a barrier. Nurses at the facility level complained that the absence of structured platforms made it impossible for them to raise concerns about the work of CHWs.^24^ The lack of communication in sharing accurate patient health information between clinics and CHWs, and between clinics and patients, was also reported as a barrier to care.^30^ For instance, when CHWs were given inaccurate information about the physical whereabouts of patients, they were unable to fulfil the task of meeting with patients and deliver services as required.^30^ This lack of communication was not only limited to clinics and CHWs, but extended to different stakeholders and structures (NGOs, clinic leadership, researchers, community leaders, etc.) in CHW programs, often with different priorities.^28,40^ Poor communication not only disrupted workflows, but also resulted in mistrust between different stakeholders involved in patient care.^30^

##### Inadequate funding

Some CHW HIV programs were donor funded and there was uncertainty about their sustainability in the long term, as governments might not be able to take over these programs when donor support ended.^38–40^ Concerns were specifically raised about CHWs creating expectations by giving patients donor-funded gifts, including financial help, material support, and food.^39^ When donors withdrew their funding, this loss of benefits could negatively affect the CHW–patient relationship.^34^

##### Inadequate monitoring

Nonperformance of assigned duties was also reported: in one study, only 15% of CHWs allocated to this task traced patients who had been lost to follow-up.^36^ Reasons given were the wide range of activities CHWs had to attend to and high patient volumes. Even more worrying is that there were no consequences for nonperformance of allocated tasks. In a study by Suri et al., and Gusdal et al., concerns about poor accountability and monitoring processes of CHW HIV programs offered by NGOs were raised.^24,37^

##### Poor integration of CHWs into the health care system

Several studies reported poor integration of community-based HIV services in clinic-based HIV treatment facilities.^26,28^ This ranged from poor integration of monthly reports on HTS performed at community and clinic levels, to an ineffective referral system between facility staff and CHWs.^30^ Issues of poor coordination of patient care between CHWs and facilities were raised, with limited platforms available for sharing patients' health information, resulting in poor and incomplete record keeping.^29,30^ On another level, there was also poor integration among CHWs, religious leaders, and traditional healers caring for PLWHIV.^26,34,45^ Some CHWs felt that the influence of traditional healers negatively impacted their efforts to retain PLWHIV in care,^45^ but there seems to be no avenues for engagement with these role players.

### Recommendations

While many different barriers were reported, most articles also provided a set of recommendations to mitigate those barriers. These recommendations are summarized in Table 3. To address stigma, recommendations were made for community-based campaigns to improve health education and deal with stigma-related issues. In addition, a move away from disease-specific approaches in favor of generalized approaches was proposed together with programs that aim to identify and address the stigma-related needs of patients.^24,26,27,35^ Professionalization of CHW programs, intensifying health campaigns, strengthening CHW supervision, making the equipment needed by CHWs available, and improving CHW attitudes were among the recommendations to improve CHW HIV services.

**Table 3.** Recommendations to Improve HIV Service Delivery by Community Health Workers in the Community

Barrier	Recommendation
Stigma	Develop campaigns to improve health education and address stigma-related issues^26^ Avoid services targeted at HIV only^27,28,47^ Identify and deal with factors leading to stigma^24^ Address stigma-related needs of patients^35^ Avoid wearing clothes that suggest provision of HIV services^28,42^
Disclosure and confidentiality	Strengthen social support^27^ Build strong patient–CHW relationships based on trust^35^ Strengthen training of CHWs about confidentiality^28^
Resources	Equip CHWs with sufficient resources needed for HIV services^24,27,45,47^ Equip CHWs with pamphlets to distribute in households^34^
Training	Recruit well-respected members of the community to be trained as CHWs^28,34^ Strengthen and standardize formal and comprehensive training focusing on confidentiality, HIV and TB-related services, and noncommunicable diseases, including mental health and substance use^28,29,34,39,44,47^
Supervision	Intensive supervision by clinical staff through individualized on-the-job mentoring^32,42^ Make use of mobile health (mHealth) tools to monitor and support CHWs^30^
Health system	Ensure that home-based HIV care builds on and integrates with existing structures^28^ Integrate CHW activities into the broader health system^28,36^ Conduct campaigns aimed at improving knowledge about HIV and the role played by CHWs^26^
Communication and reporting	Introduce sustainable and safe mobile health (mHealth) platforms to improve the quality of data collected by CHWs, communication, and reporting.^30^ Involve patients, in particular PLWHIV, in designing such platform^30^ Strengthen the links between community- and facility-based health services^25^ Integrate CHW programs into the primary health care system and involve all other stakeholders caring for PLWHIV, including traditional healers and religious leaders^28,36,48^
Funding	Create more sustainable ways of funding CHW programs^38^
Performance	Introduce ongoing assessment of CHW performance and attitudes toward patients^36^ Develop standard protocols to guide CHWs on activities, workflow, triaging of patients, accountability, and performance monitoring^24,36^

CHW, Community Health Worker; HIV, human immunodeficiency virus; PLWHIV, people living with HIV; TB, tuberculosis.

## Discussion

CHWs are a very important cadre of health care workers since they have a privileged vantage point into the community. They have more time to interact with PLWHIV, influence how PWHIV engage with care and access resources, are familiar with the environment and socioeconomic reality they live in, and are more able to interact with family members when there is a need.^48,49^ However, some authors feel that CHWs are currently underutilized in providing patient support and may not be producing observable benefits.^50^ This systematic, aggregated, narrative review on the roles, barriers, and recommendations for CHWs with regard to community-based HIV care support this view but also found that barriers and challenges remain that have to be addressed for patients and households to derive the maximum benefit from such services.

Our findings on the roles of CHWs support those from another systematic review performed by Mwai et al.^16^ Some important differences in our article include CHWs offering HTS outside facility settings as well as CHWs providing psychosocial support. Additionally, we found that CHW activities in caring for PLWHIV also included nontraditional home-based care activities for severely ill patients, such as cooking, feeding, cleaning, changing soiled linen, and fetching water.^32,39,51^ While these activities might not be viewed to be part of CHWs' scope of practice, many PLWHIV, especially child-headed households, require them as some have suffered multiple crises and structural injustices, including chronic poverty, recurrent illnesses, weak social ties, deceased family members, and little or no ability to generate an income.^48^ Due to their ground-level interaction with communities, well-functioning CHW programs are better placed to address these needs of PLWHIV by liaising between community role players (including clinics, social services, government departments, NGOs, academic institution, etc.) and PLWHIV.^24^

Additional activities also included the delivery of medication, which has been shown to improve patient satisfaction with CHW services,^52^ since it mitigates some of the reasons patients miss ART clinic visits, such as lack of time, transport costs, and long waiting times.^53,54^ This has proven to be critical during the coronavirus disease pandemic when traditional health services were severely disrupted and congestion at health facilities posed the risk of extensive spread of the disease.^55^

In South Africa, there are 5.2 million people accessing ART, accounting for 70% of PLWHIV.^1^ To reduce travel cost, waiting times, and clinic congestion while concurrently enhancing patient adherence and satisfaction, the Centralized Chronic Medicine Dispensing and Distribution (CCMDD) program was implemented in South Africa in 2014.^56^ While this intervention has been effective in decongesting clinics,^57,58^ it faces many challenges such as inadequate feedback about patients not picking up their medication as well as the absence of clinical monitoring and adherence support.^59^ The delivery of ART by CHWs to patients' homes could be a valuable alternative since it includes monitoring and support but, notwithstanding the benefits, also presents numerous challenges. These are not only logistical, such as incorrect delivery addresses, recipients not being at home, and the need for deliveries outside of CHW catchment areas, but are also of an ethical nature.^28,60^ Serious consideration should be given to unequal distribution of services, risk of inadvertent disclosure, and whether such services may perpetuate internalized stigma and even prevent patients from receiving monitoring and counseling from trained medical professionals.

In that regard, it is positive to note that the results of this study suggest that the roles of CHWs are evolving toward more medicalized services, such as treatment support with the aim improving adherence to ART.^26^ CHWs are therefore well positioned to help programs achieve the UNAIDS 2030 goals, and countries should be encouraged to actively incorporate them in their planning. Unfortunately, in most SSA countries, CHWs are not well equipped with sufficient and current HIV-related knowledge to offer accurate education about HIV and ART.^26,28,61^ To adequately deliver these essentials, and in many respects, medicalized services, and meet the demands of PLWHIV and HIV programs, CHWs require more training situated within a professionalized approach. We argue that CHWs should no longer be viewed as just a temporary intervention, but as allied health care professionals in their own right and, as such, undergo structured and well-coordinated certified training with specific core competencies that address the needs of the communities in which they work.^62^

Our findings reveal several, multilevel barriers related to community-based HIV services that should be considered in all SSA (and arguably in all) CHW programs. One of the most important barriers was persistent and prevailing HIV-related stigma, which leads to nondisclosure of HIV status to CHWs, often secondary to a perceived lack of confidentiality. It is well known that stigma can affect access to health care, medication adherence, social interactions, and social support.^63^ In this systematic review, it was clear that stigma and fear of stigmatization were some of the barriers that could hinder the delivery of health services by CHWs to PLWHIV. Both external and internalized stigma still play a significant role in the lives of PLWHIV, with some PLWHIV being excluded from social events, gossiped about, teased, and insulted. This affects their willingness to receive community-based HIV care.^28,64^ These findings are corroborated by previous studies that demonstrated that the fear of HIV-related stigma resulted in nonuptake of HIV services.^65,66^ To effectively deal with perceived and internalized stigma, it is important to understand the extent of this problem using tools such as validated HIV stigma indices.

In a Stigma Index 2.0 study conducted in Cameroon, Senegal, and Uganda, HIV-related external stigma was experienced by more than one-third of respondents.^67^ Similarly, in a South African stigma index study of 10,473 PLWHIV, 35.5% of participants reported experiencing some external stigma.^64^ Key populations groups, such as sex workers and men who have sex with men, experience the highest levels of stigma,^68^ often as a result of moral judgment.^69^ It has also been reported that health workers with lower educational levels, such as CHWs, hold more judgmental beliefs.^68^ Comprehensive planning and implementation strategies targeted at stigma reduction within community settings and among CHWs are needed and should also focus on key populations.^68^

The introduction of CHW programs in most SSA countries was closely linked to HIV services, resulting in CHW activities being associated with being HIV positive and potential social rejection and discrimination of the recipients of such programs.^28,66^ This association is reinforced when PLWHIV who have physical manifestations of HIV and AIDS are regularly visited by CHWs, hence perpetuating the risk of social isolation and exclusion of recipients.^36^ To address this, there is a need to move away from programs that focus on HIV-specific care only, toward programs that deliver integrated health services; thereby addressing the needs of all household members and not just those of PLWHIV.^45^ This could also have additional benefits, such as strengthening of community-based noncommunicable disease programs, women's health, including contraception, and improved child health programs.

A review of the impact of physical environmental factors on patients in health facilities by Huisman et al. highlights how the physical environment can impede privacy, confidentiality, the healing process, and the well-being of patients.^70^ The average household size in SSA was 6.9 people per household in 2019, which was the largest average household size recorded worldwide.^71^ With almost seven people per household, it is difficult to maintain the privacy and confidentiality that this review suggests is needed for disclosure of HIV status and acceptance of home-based HIV services.^35^ This suggests that serious reconsideration of the household as the preferred site of CHW interactions is warranted. For CHWs to prioritize privacy and confidentiality for PLWHIV with this specific need, they should be empowered to make arrangements to meet with PLWHIV in areas where privacy and confidentiality can be maintained.

Other barriers related to the lack of support of CHWs in terms of resources, training, stakeholder assistance, communication channels, funding, and monitoring. Some of these resources, such as educational material, are already in existence, and need to be distributed effectively in conjunction with structured in-service training on these materials for CHWs. It has been shown that educational pamphlets not only improve the health literacy of community members but also the evidence-based health knowledge of CHWs.^72^ Appropriate, sufficient, and understandable educational resources will therefore help ensure that CHWs offer better-quality HIV services. Significant effort should be made to address the lack of oversight, structured support for challenging cases, and incentives to promote a high quality of home-based HIV services.^50^

Poor communication and linkage between many stakeholders offering HIV services could be partially addressed through the introduction of mobile (mHealth) technology platforms. In recent years, there has been a shift toward technology-based approaches aimed at improving HIV service delivery, including improving patients' HIV literacy, improving linkage to care, retention in care, and treatment adherence.^73^ A systematic review by Braun et al. reported that mHealth improved the quality of services provided by CHWs.^74^ These mHealth interventions could assist in improve reporting, communication, supervision, guide learning, improve linkage to care, and keep confidential information secured through user passwords.^74,75^

Finally, from our review, it is clear that the sustainability of CHW programs is still a challenge, with many CHW programs relying on donor support.^26,34^ When funding comes to an end, this can severely disrupt patient care and affect CHW–patient relationships. There is evidence that outcomes improve when PLWHIV receive continuous care from CHWs.^16^ Recently, the role of CHWs have successfully been expanded to the coronavirus disease pandemic, especially in poor countries, where CHWs were involved in contact tracing, coronavirus disease-related education, screening, and monitoring of patients.^76^ Vertical, facility-based health care services are unable to deal with the growing burden of disease in the populations they serve.^55^ To appropriately strengthen health care systems for this task, it seems opportune for governments to commit to supporting CHW programs and invest the resources needed to successfully offer integrated health care services at a household level.

In conclusion, given the large HIV burden in SSA, CHWs function as a critical link between the community and health facilities. Our review found that CHWs are providing treatment support together with home-based care (HBC) services. There is however a gap in training, resources, and supervision. To strengthen CHW programs, substantial improvements are needed. The most urgent are addressing HIV-related stigma, introducing updated and relevant CHW training, strengthening the supervision of CHWs, coordinating care between the home and facilities, incorporating patient-centered mHealth approaches, and committing to avail funding and the resources needed for successful community-based care.

## Supplementary Material

Supplemental data
