# Supplementary material for: Roles, Barriers, and Recommendations for Community Health Workers Providing Community-Based HIV Care in Sub-Saharan Africa: A Review
Source: AIDS Patient Care STDS. 2022 Apr 14;36(4):130–44. doi: 10.1089/apc.2022.0020 (PMC9057893; doi:10.1089/apc.2022.0020)
Supplement: Supplemental data [file Supp_TableS1.docx]

**Supplementary Table 1: Quality of studies**

| **References** | **^35^** | **^29^** | **^24^** | **^34^** | **^25^** | **^27^** | **^28^** | **^30^** | **^38^** | **^26^** | **^44^** | **^32^** | **^42^** | **^36^** | **^37^** | **^39^** | **^33^** |
| --- | --- | --- | --- | --- | --- | --- | --- | --- | --- | --- | --- | --- | --- | --- | --- | --- | --- |
| Are the research questions clear? (1) | 1 | 1 | 1 | 1 | 0.5 | 1 | 1 | 1 | 1 | 1 | 1 | 1 | 1 | 1 | 1 | 1 | 1 |
| Are the research questions suited to qualitative inquiry (1) | 1 | 1 | 0.5 | 1 | 1 | 1 | 1 | 1 | 1 | 1 | 1 | 1 | 1 | 1 | 1 | 1 | 1 |
| Are the following clearly described? (1)   - Sampling - Data collection - analysis | 1 | 1 | 1 | 1 | 1 | 1 | 1 | 1 | 1 | 1 | 1 | 1 | 0.5 | 1 | 1 | 1 | 1 |
| Are the claims made supported by sufficient evidence (1) | 1 | 1 | 1 | 1 | 1 | 1 | 1 | 1 | 1 | 1 | 1 | 1 | 1 | 1 | 1 | 1 | 1 |
| Are the data, interpretations and conclusions clearly integrated? (1) | 1 | 1 | 1 | 1 | 1 | 1 | 1 | 1 | 1 | 1 | 1 | 1 | 1 | 1 | 1 | 1 | 1 |
| Does the paper make a useful contribution? (1) | 1 | 1 | 1 | 1 | 1 | 1 | 1 | 1 | 1 | 1 | 0.5 | 1 | 1 | 1 | 1 | 1 | 1 |
| How relevant is this study to this review (1) | 4 | 4 | 4 | 4 | 2 | 3 | 4 | 3 | 2 | 4 | 3 | 4 | 4 | 4 | 4 | 4 | 4 |
